# The complete genome sequence of the methanogenic archaeon ISO4-H5 provides insights into the methylotrophic lifestyle of a ruminal representative of the *Methanomassiliicoccales*

**DOI:** 10.1186/s40793-016-0183-5

**Published:** 2016-09-06

**Authors:** Yang Li, Sinead C. Leahy, Jeyamalar Jeyanathan, Gemma Henderson, Faith Cox, Eric Altermann, William J. Kelly, Suzanne C. Lambie, Peter H. Janssen, Jasna Rakonjac, Graeme T. Attwood

**Affiliations:** 1 AgResearch Limited, Grasslands Research Centre, Palmerston North, New Zealand; 2 Institute of Fundamental Sciences, Massey University, Palmerston North, New Zealand

**Keywords:** Methanogen, Methane, Ruminant, *Methanomassiliicoccales*, Pyrrolysine

## Abstract

Methane emissions from agriculture represent around 9 % of global anthropogenic greenhouse emissions. The single largest source of this methane is animal enteric fermentation, predominantly from ruminant livestock where it is produced mainly in their fermentative forestomach (or reticulo-rumen) by a group of archaea known as methanogens. In order to reduce methane emissions from ruminants, it is necessary to understand the role of methanogenic archaea in the rumen, and to identify their distinguishing characteristics that can be used to develop methane mitigation technologies. To gain insights into the role of methylotrophic methanogens in the rumen environment, the genome of a methanogenic archaeon has been sequenced. This isolate, strain ISO4-H5, was isolated from the ovine rumen and belongs to the order *Methanomassiliicoccales*. Genomic analysis suggests ISO4-H5 is an obligate hydrogen-dependent methylotrophic methanogen, able to use methanol and methylamines as substrates for methanogenesis. Like other organisms within this order, ISO4-H5 does not possess genes required for the first six steps of hydrogenotrophic methanogenesis. Comparison between the genomes of different members of the order *Methanomassiliicoccales* revealed strong conservation in energy metabolism, particularly in genes of the methylotrophic methanogenesis pathway, as well as in the biosynthesis and use of pyrrolysine. Unlike members of *Methanomassiliicoccales* from human sources, ISO4-H5 does not contain the genes required for production of coenzyme M, and so likely requires external coenzyme M to survive.

**Electronic supplementary material:**

The online version of this article (doi:10.1186/s40793-016-0183-5) contains supplementary material, which is available to authorized users.

## Introduction

Ruminant animals have evolved a digestive system in which microbes in their rumen break down plant fiber and provide fermentation end-products and other nutrients for growth and development of the animal [1]. The rumen is densely populated with bacteria, archaea, ciliate protozoa, anaerobic fungi and viruses which participate in complex interactions to bring about the digestion of forage material. The archaeal community is made up almost exclusively of methanogens, which use simple energy sources such as hydrogen, formate and methyl compounds and produce methane. Rumen methanogens play an important role in preventing the accumulation of hydrogen derived from microbial fermentation of plant polysaccharides. This allows reduced cofactors, generated during microbial fermentation, to be re-oxidised so that the main fiber-degrading function of the rumen can continue. The methane formed from this process is belched from the animal to the atmosphere, where it contributes a global warming potential (over 100 years, GWP_100_) of around 34× that of carbon dioxide [2, 3]. The production of methane represents a loss of energy from the ruminant, and depending on the diet, this loss can represent 3.8 to 12.8 % of energy contained in the diet [4–6].

Methanogens are classified into three broad categories based on the compounds they use for methanogenesis: hydrogenotrophic, methylotrophic and acetoclastic [7]. In the rumen, methane is formed mainly via the hydrogenotrophic and methylotrophic pathways. Members of the new order of methanogenic archaea, *Methanomassiliicoccales*, are hydrogen-dependent methylotrophic methanogens and have been detected in various habitats, including landfills, rice fields, marine thermal vents, fresh water, and in the digestive tracts of termites, millipedes, chickens, ruminants and humans [8–18]. The *Methanomassiliicoccales* are considered to be an important group in the rumen environment and were originally referred to as Rumen Cluster C methanogens [19, 20]. Their abundance in the rumen is highly variable, according to 16*S* ribosomal RNA gene surveys [21–23], but on average, they are the second most abundant order of rumen methanogens and constitute around 16 % of the rumen archaeal community based on clone library analyses [24], and 13 % of rumen archaeal community based on pyrosequencing [25]. Representatives of these organisms have only recently been isolated in culture, and genomic information on members of the *Methanomassiliicoccales* are available only for isolates from human, bovine [26–29] and termite sources (NCBI Reference Sequence: NC_020892.1). This study reports the complete genome sequence of an ovine rumen member of *Methanomassiliicoccales*, designated methanogenic archaeon ISO4-H5.

## Organism information

### Classification and features

**Fig. 1:**
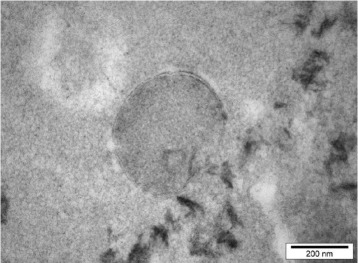
Transmission electron micrograph of negatively stained thin section of the methanogenic archaeon ISO4-H5. The sample was prepared as previously described [60]. Images were captured using a Philips CM10 Transmission Electron Microscope, using an Olympus SIS Morada camera and SIS iTEM software (Germany)

**Fig. 2:**
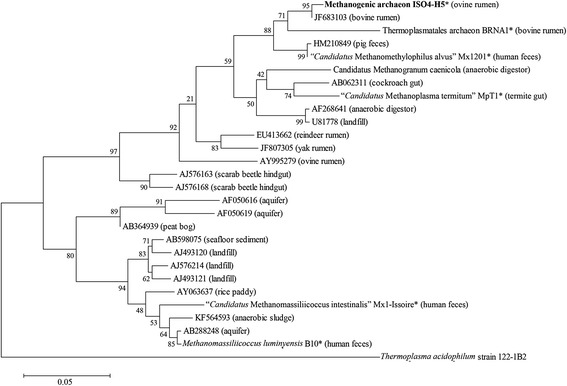
Phylogenetic analysis of *Methanomassiliicoccales* 16*S* rRNA gene sequences. Phylogenetic tree showing the relationships of methanogenic archaeon ISO4-H5 (shown in bold print) relative to other type and non-type strains within the order *Methanomassiliicoccales*. The phylogeny was inferred from 16*S* rRNA gene nucleotide sequences (1474 bp internal region) aligned using the Maximum Likelihood method based on the Kimura 2-parameter model [61]. Evolutionary analyses were conducted in MEGA6 [62]. The bootstrap consensus tree from 1000 replicates [63] was used to infer the evolutionary history of the taxa analysed. Bar: 0.05 substitutions per nucleotide position. The GenBank accession numbers of environmental sequences are displayed with the source habitat given in brackets. Strains whose genomes have been sequenced are marked with an asterisk. The initial tree for the heuristic search was obtained by applying the Neighbor-Joining method to a matrix of pairwise distances estimated using the Maximum Composite Likelihood (MCL) approach. All positions containing gaps or missing data were eliminated, giving a total of 455 positions in the final dataset. The 16*S* rRNA gene sequence from *Thermoplasma acidophilum* 122-1B2 was used as an outgroup

A methane-forming enrichment culture was originally obtained from a 9-year-old Romney wether sheep in New Zealand grazing a ryegrass-clover pasture diet [30]. The enrichment culture contained the methanogenic archaeon, ISO4-H5, and a Gram-negative bacterium, subsequently identified as being closely related to *Succinivibrio**dextrinosolvens* and designated as strain H5. The methanogenic archaeon ISO4-H5 grows slowly and requires 3 to 4 days to generate detectable methane in the culture headspace. The optical density of cultures after maximal methane formation is very low and ISO4-H5 cells cannot be visualized *via* fluorescence microscopy at 420 nm due to the apparent lack of the fluorescent 8-hydroxy-5-deazaflavin cofactor, known as F_420_ [30]. The organism has only a thin bi-layer cell membrane, and no S-layer or cell wall was observed in electron micrographs of thin sections of ISO4-H5 cells (Fig. 1). The 16*S* ribosomal RNA gene of ISO4-H5 is 96 % identical to “*Candidatus* Methanomethylophilus alvus” Mx1201 enriched from human feces [27], and 95 % identical to *Thermoplasmatales* archaeon BRNA1 enriched from bovine rumen (Fig. 2). All three are members of the order *Methanomassiliicoccales*, but potentially each represent different species [31]. The general features of methanogenic archaeon ISO4-H5 are shown in Table 1 and Additional file 1: Table S1.

**Table 1:** Classification and general features of the methanogenic archaeon ISO4-H5

MIGS ID	Property	Term	Evidence code^a^
	Current classification	Domain: *Archaea*	TAS [64]
		Phylum: *Euryarchaeota*	TAS [65]
		Class: *Thermoplasmata*	TAS [66]
		Order: *Methanomassiliicoccales*	TAS [66, 67]
		Family:	
		Genus:	
		Species:	
		Strain: ISO4-H5	TAS [30]
	Gram stain	Not applicable	
	Cell shape	Coccoid 0.3 μm ~ 0.6 μm diameter	
	Motility	Non-motile	
	Sporulation	Not spore-forming	IDA
	Temperature range	Not reported	
	Optimum temperature	38/39 °C	TAS [30]
	pH range	Not reported	
	Optimum pH	Not reported	
	Carbon source	Not reported	
	Energy source	H_2_ + methanol, mono-, di-, or trimethylamine	IDA
	Terminal electron receptor	Methyl-substrates	IDA
MIGS-6	Habitat	Ovine rumen	TAS [30]
MIGS-6.3	Salinity	Not reported	
MIGS-22	Oxygen	Strict anaerobe	IDA
MIGS-15	Biotic relationship	Symbiont of ruminants	TAS [30]
MIGS-14	Pathogenicity	Not known as a pathogen	NAS
MIGS-4	Geographic location	Palmerston North, New Zealand	IDA
MIGS-5	Sample collection time	Autumn, 2008	IDA
MIGS-4.1	Latitude	Latitude: -40.35 (40°21'00"S)	IDA
MIGS-4.2	Longitude	Longitude: +175.61 (175°36'36"E)	IDA
MIGS-4.4	Altitude	30 m	IDA

^a^Evidence codes – *TAS* Traceable Author Statement (i.e., a direct report exists in the literature), *IDA* Inferred from Direct Assay, *NAS* Non-traceable Author Statement (i.e., not directly observed for the living, isolated sample, but based on a generally accepted property for the species, or anecdotal evidence). These evidence codes are from the Gene Ontology project [68]

## Genome sequencing information

### Genome project history

To gain insight into the role of methylotrophic methanogens in the rumen environment, the genome of the methanogenic archaeon isolate ISO4-H5 was sequenced. Methanogenic archaeon isolate ISO4-H5 represents the first genome sequence of a member of the order *Methanomassiliicoccales* isolated from the ovine rumen. A summary of the genome project information is shown in Table 2.

**Table 2:** ISO4-H5 genome project information

MIGS ID	Property	Term
MIGS-31	Finishing quality	High-quality, closed genome
MIGS-28	Libraries used	454 3 kb mate paired-end library
MIGS-29	Sequencing platforms	454 GS-FLX Titanium chemistry
MIGS-31.2	Fold coverage	43.8× (454)
MIGS-30	Assemblers	Newbler
MIGS-32	Gene calling method	GLIMMER2.02 + BLASTX [69]
	Locus Tag	AR505
	Genbank ID	CP014214
	Genbank Date of Release	12-February-2016
	GOLD ID	Gp0125684
	BIOPROJECT	PRJNA292473
	BIOSAMPLE	SAMN03976563
MIGS 13	Source Material Identifier	Methanogenic archaeon ISO4-H5
	Project relevance	Ruminant methane emissions

### Growth conditions and genomic DNA preparation

The initial enrichment cultures were obtained by inoculation of sheep rumen contents into BY medium [32] upplemented with (final concentrations), SL10 trace elements solution (1 mL/L) [33], selenite/tungstate solution (1 mL/L) [33], sodium acetate (20 mM), sodium formate (60 mM), methanol (20 mM), vitamin 10 solution (0.1 ml per 10 mL culture tube) [32], and coenzyme M (CoM) (10 μM) [34]. The last two additives were added to the sterilized medium from filter-sterilized stock solutions. Hydrogen (H_2_) was supplied as the energy source by pumping the culture vessels to 180 kPa over pressure with an 80:20 mixture of H_2_: carbon dioxide (CO_2_). ISO4-H5 was enriched in tubes receiving sheep rumen contents diluted by a factor of 16,384,000 [30]. Several approaches were used to reduce the bacteria in the enrichment culture, including a 10-fold dilution, the addition of antibiotics (combinations of streptomycin, ampicillin, bacitracin at 10 μg/mL each, and vancomycin at 86.7 μg/mL), heat treatment of the enrichment culture at 50 °C for 10 to 30 min, and application of lysozyme (2.5 mg/mL). These approaches produced a limited diversity enrichment culture containing ISO4-H5 and *S. dextrinosolvens* H5, which was verified by phase contrast epifluorescence microscopy and bacterial and archaeal 16*S* rRNA gene sequencing. Genomic DNA was extracted from cells harvested from a freshly grown (7 d incubation time) 2 L enrichment culture using a modified version of a liquid N_2_ freezing and grinding method [35], in which treatment with 2.5 mg lysozyme/mL and 0.8 mg proteinase K/mL replaced the 1 % w/v sodium dodecyl sulfate step, before a Genomic-tip 500/G (Qiagen, Germany) was used, following the manufacturer’s instructions, in place of the phenol/chloroform extraction steps.

### Genome sequencing and assembly

The DNA extracted from the ISO4-H5 enrichment culture was sequenced *via* pyrosequencing of a 3 kb mate paired-end sequence library using the 454 GS FLX platform with Titanium chemistry (Macrogen, Korea). Pyrosequencing reads provided 43.8× coverage of the combined ISO4-H5 and *Succinivibrio**dextrinosolven*s H5 genomes, and were assembled using the Newbler assembler version 2.7 (Roche 454 Life Sciences, USA). The Newbler assembly resulted in 176 *Succinivibrio**dextrinosolvens* H5 contigs across 28 scaffolds and 47 ISO4-H5 contigs in a single scaffold. The assignment of scaffolds to genomes was based on G + C content analysis and identification of the methanogenesis marker gene, methyl coenzyme M reductase (*mrtA*). Sequence gap closure was managed using the Staden package [36] and gaps were closed using standard PCR techniques with Sanger sequencing. A total of 163 additional sequencing reactions were used to close gaps and to improve the quality of the genome sequence, ensuring correct assembly and to resolve base conflicts.

### Genome annotation

Genome annotation was carried out as previously described [34, 37] and the ISO4-H5 genome sequence was prepared for NCBI submission using Sequin [38]. The guanosine residue of the start codon of the Cdc6-1 replication initiation protein gene (AR505_0001) was chosen as the first base for the ISO4-H5 genome. The nucleotide sequence of the ISO4-H5 chromosome has been deposited in Genbank under accession number CP014214.

## Genome properties

The genome of ISO4-H5 consists of a single, 1,937,882 bp, circular chromosome with a G + C content of 54 %. A total of 1,817 protein-coding genes were predicted, representing 90.2 % of the total genome sequence. A Cluster of Orthologous Groups category was assigned to 1,434 of the protein-coding genes, and the properties of the genome are summarized in Tables 3 and 4.

**Table 3:** ISO4-H5 genome nucleotide content and gene count

Attribute	Value	% of total^a^
Genome size (bp)	1,937,882	100.00
DNA coding (bp)	1,747,977	90.20
G + C content (bp)	1,046,533	54.0
DNA scaffolds	1	100.00
Total genes	1,874	100.00
Protein-coding genes	1,817	96.95
RNA genes	54	2.29
Pseudo genes	3	0.16
Genes in internal clusters	NA	
Genes with function prediction	1113	59.39
Genes assigned to COGs	1,434	76.52
Genes with Pfam domains	396	21.13
Genes with signal peptides	157	8.38
Genes with transmembrane helices	352	18.78
CRISPR repeats	1	

^a^Total is based on either the size of the genome in base pairs, or the total number of protein coding genes in the annotated genome

**Table 4:** ISO4-H5 genes assigned to COG functional categories

Code	value	% of total^a^	Description
J	144	7.89	Translation
A	1	0.05	RNA processing and modification
K	67	3.67	Transcription
L	123	6.74	Replication, recombination and repair
B	1	0.05	Chromatin structure and dynamics
D	9	0.49	Cell cycle control, mitosis and meiosis
Y	0	0.00	Nuclear structure
V	17	0.93	Defense mechanisms
T	20	1.10	Signal transduction mechanisms
M	27	1.48	Cell wall/membrane biogenesis
N	2	0.11	Cell motility
Z	0	0.00	Cytoskeleton
W	0	0.00	Extracellular structures
U	13	0.71	Intracellular trafficking and secretion
O	53	2.90	Posttranslational modification, protein turnover, chaperones
C	87	4.77	Energy production and conversion
G	40	2.19	Carbohydrate transport and metabolism
E	99	5.42	Amino acid transport and metabolism
F	46	2.52	Nucleotide transport and metabolism
H	105	5.75	Coenzyme transport and metabolism
I	18	0.99	Lipid transport and metabolism
P	90	4.93	Inorganic ion transport and metabolism
Q	14	0.77	Secondary metabolites biosynthesis, transport and catabolism
R	242	13.26	General function prediction only
S	126	6.90	Function unknown
-	481	26.35	Not in COGs

^a^The total is based on the total number of protein coding genes in the annotated genome

**Fig. 3:**
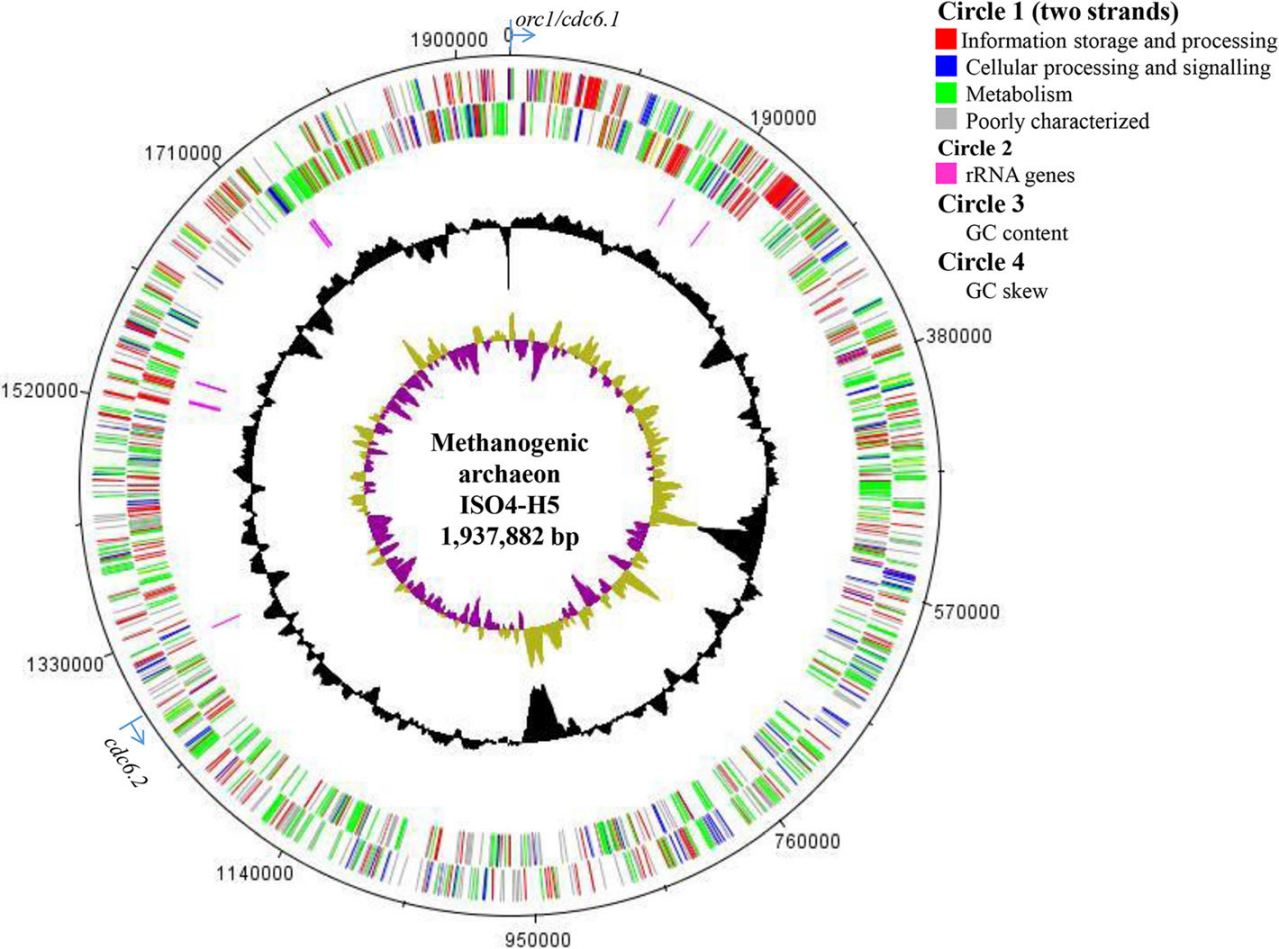
Circular representation of ISO4-H5 genome. Circles are referred to as 1 (outermost) to 4 (innermost). Circle 1: predicted ORFs on the positive (+) and negative (–) strands, respectively. ORFs are colored based on the Clusters of Orthologous (COG) categories. Circle 2: location of the rRNA genes. Circle 3: %G + C content. Circle 4: GC bias [(G − C)/(G + C)], khaki indicates values >1, purple <1. This image was generated with DNA plotter [70]

ISO4-H5 is predicted to contain two Cdc6 genes. Cdc6.1 (AR505_0001) is adjacent to two origin recognition box (ORB) motifs downstream [39], while Cdc6.2 (AR505_1205) is located 661 kb away from the Cdc6.1 gene and is not associated with any ORB motif. Therefore, Cdc6.1 is predicted to be the origin of replication for ISO4-H5 (Fig. 3). The presence of multiple origins of replications is a feature also observed in the genome sequences of other members of *Methanomassiliicoccales*, including BRNA1 (TALC00001, TALC00716, 645 kb apart), Mx1201 (MMALV_00010, MMALV_10400, 637 kb apart), Mx1-Issoire (H729_00005, H729_08750, 90 kb apart), and B10 (WP_019178385, WP_019178317). The ISO4-H5 genome contains genes predicted to be integrases (AR505_0313, 0669, 0931, 1543, 1570, 1640, 1697), as well as several Clustered Regularly Interspaced Short Palindromic Repeat (CRISPR) genes (AR505_1089 – 1095) associated with a CRISPR region containing 35 repeats (bases 1,153,894 to 1,155,995). There is evidence of a mobile element in the ISO4-H5 genome (AR505_0313–AR505_0358) which excised and segregated from the chromosome over several passages between the sequencing of the genome and subsequent analyses of the annotated locus. The 32 kb mobile element harbors 37 hypothetical protein genes, three adhesin-like protein genes, three DNA-cytosine methyltransferase genes, one phage integrase gene, one DNA mismatch endonuclease gene and one Membrane Occupation and Recognition Nexus (MORN) repeat-containing protein [40]. No plasmids were identified in the ISO4-H5 genome. The genome contains a predicted toxin/antitoxin module (AR505_0857, 0858) and a death-on-curing family protein (AR505_1566), although the latter lacks an identifiable gene encoding a partner toxin [41, 42].

## Insights from the genome

**Fig. 4:**
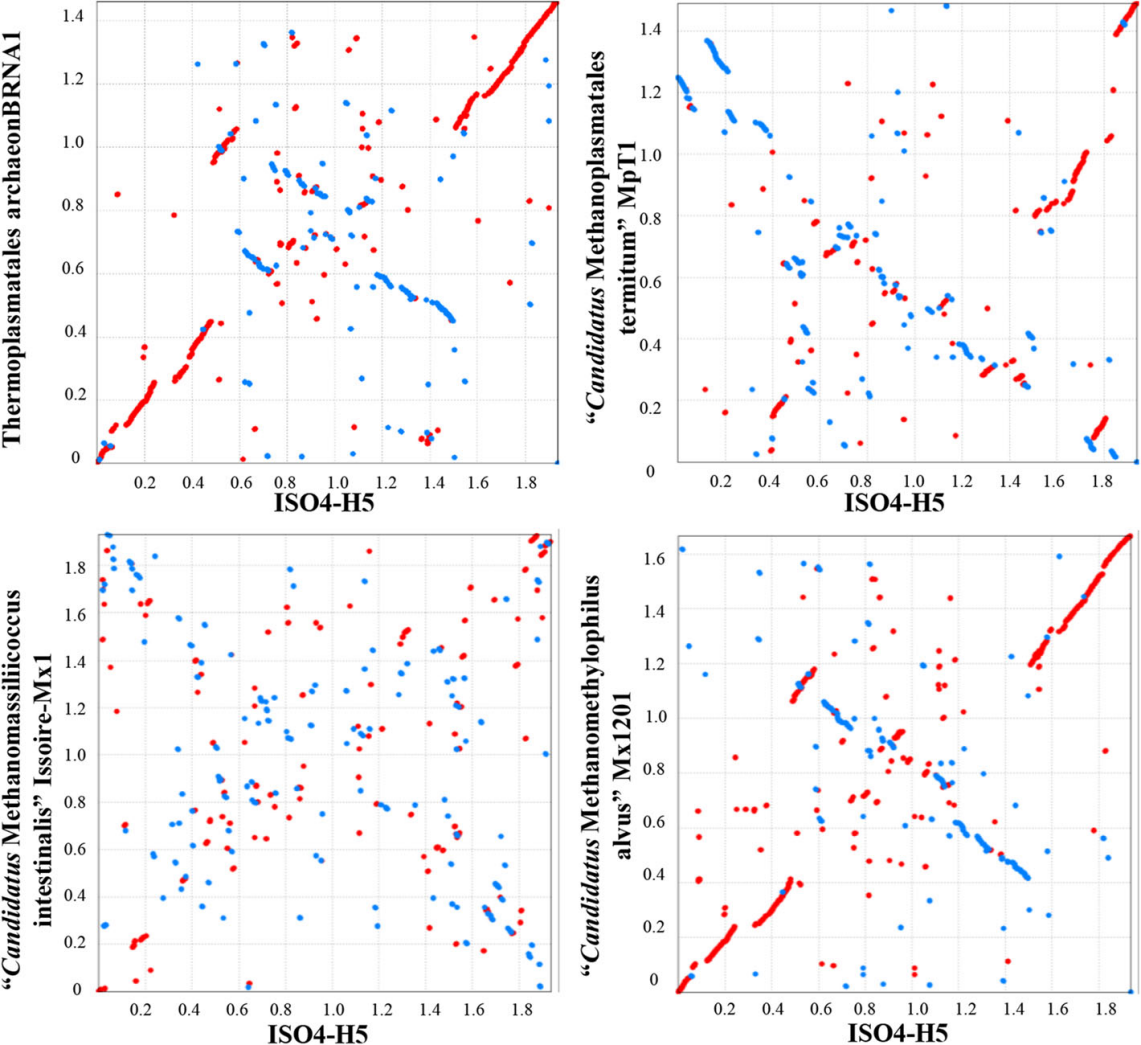
Gene synteny plots for genomes of members of the order *Methanomassiliicoccales*. PROmer alignments of the ISO4-H5 genome against completed genomes from members of *Methanomassiliicocaccales* are shown. The alignments were plotted using MUMmer [71] with forward matches shown in red and reverse matches in blue. The units displayed on both axes are in million base pairs

The genomes of several members of *Methanomassiliicoccales* are publically available, including *M. luminyensis* B10 isolated from a human source, “*Candidatus* Methanomethylophilus alvus” Mx1201 and “*Candidatus* Methanomassiliicoccus intestinalis” Mx1-Issoire enriched from human sources, “*Candidatus* Methanoplasma termitum” MpT1 enriched from termite gut, and *Thermoplasmatales* archaeon BRNA1 enriched from the bovine rumen. These genomes were compared with ISO4-H5 (Table 5). ISO4-H5 is very similar in genome size to the other members of *Methanomassiliicoccales*, with *M. luminyensis* B10 being the exception, with a genome 35 % larger than ISO4-H5. The genomic G + C content of the *Methanomassiliicoccales* range from 49 to 60 %, with “*Candidatus* Methanomassiliicoccus intestinalis” Mx1-Issoire being different to the rest with a genomic G + C content of 41 %. The organization of genes within the ISO4-H5 genome shows best synteny with “*Candidatus* Methanomethylophilus alvus” Mx1201 and *Thermoplasmatales* archaeon BRNA1 (Fig. 4), its two closest genome-sequenced relatives.

**Table 5:** Genomes of members of *Methanomassiliicoccales* from rumen and human sources

Species	Status	Isolation source	Genome size (Mb)	Accession #	CDS	% GC	Reference
Methanogenic archaeon ISO4-H5	Complete	Ovine rumen	1.94	CP014214	1,823	54	This report
*Candidatus* Methanomassiliicoccus intestinalis Mx1-Issoire	Complete	Human feces	1.93	CP005934	1,876	41	[26]
*Candidatus* Methanomethylophilus alvus Mx1201	Complete	Human feces	1.67	CP004049	1,700	56	[27]
*Methanomassiliicoccus luminyensis* B10	Draft	Human feces	2.62	CAJE01000001 – CAJE-1000026	2,669	60	[28]
*Candidatus* Methanoplasma termitum MpT1	Complete	Termite gut	1.49	CP010070	1,415	49	[29]
*Thermoplasmatales* archaeon BRNA1	Complete	Bovine rumen	1.46	CP002916	1,577	58	Unpublished

### Methanogenesis

**Fig. 5:**
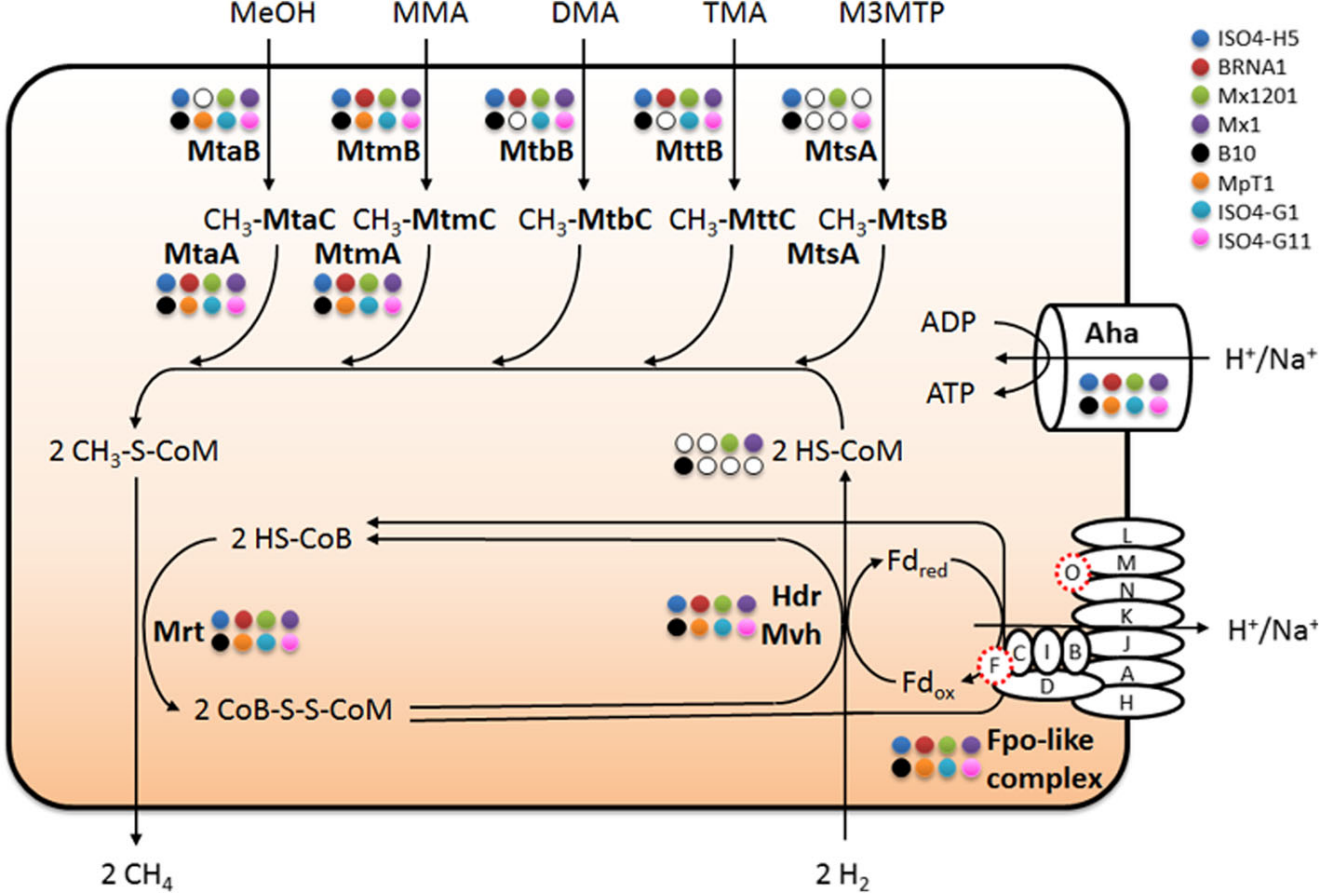
The proposed methanogenesis pathway in ISO4-H5 growing with hydrogen and methanol, mono-, di-, tri-methylamine, or methyl-3-methylthiopropionate. Two methyl groups are needed from the methyl donors for every two methane formed. Methyl groups from methanol (MeOH), monomethylamine (MMA), dimethylamine (DMA), trimethylamine (TMA) and methyl-3-methylthiopropionate (M3MTP) are transferred onto methyl-binding corrinoid proteins CH_3_-MtaC, CH_3_-MtmC, CH_3_-MtbC, CH_3_-MttC and CH_3_-MtsB by specific corrinoid methyl transferases MtaB, MtmB, MtbB, MttB and MtsA respectively. The methyl groups are then transferred from the corrinoid proteins to CoM by CoM methyltransferase MtaA, MtmA, and bifunctional MtsA. Methyl-CoM is reduced to methane by the methyl coenzyme M reductase Mrt complex with cofactor HS-CoB. Heterodisulfide reductase complex Hdr and hydrogenase complex Mvh couple electron bifurcation to cofactor regeneration, and are coupled to the Fpo-like complex to generate a membrane potential for ATP production. The H^+^ (or Na^+^) ratio to ATP is not known, and the reconstruction of the pathway is based on the schemes proposed by Lang *et al*. [28]. The presence or absence of each gene or the complete pathway for coenzyme M synthesis, in members of *Methanomassiliicoccales* is highlighted by colored circles; a white circle indicates absence in a genome. The *fpo*F and *fpo*O genes that are not found in members of *Methanomassiliicoccales* but exist in *M. barkeri* are represented by dotted red circles in the Fpo-like complex

Members of the order *Methanomassiliicoccales* rely solely on hydrogen-dependent methylotrophic methanogenesis to produce energy. However, they use only part of the pathway reported for other methylotrophic methanogens (Fig. 5), such as members of the genera *Methanosarcina* and *Methanosphaera* [43, 44]. *Methanosarcina* spp. disproportionate methanol by electron bifurcation, oxidizing one mole to produce CO_2_ while generating reducing potential to reduce three further moles to methane. The methanogenesis pathway in ISO4-H5 lacks the genes encoding the enzymes required to oxidize methanol to CO_2_, and is predicted to only reduce methylated compounds directly to methane. Functionally, this is similar to *Methanosphaera stadtmanae* MCB-3, which encodes all the genes for the enzymes needed to oxidize methanol to CO_2_ but does not use this pathway due to the lack of genes encoding synthesis of molybdopterin, a cofactor required for formation of an active formylmethanofuran dehydrogenase [44]. ISO4-H5 is predicted to use a heterodisulfide reductase (HdrABC) and a methyl-viologen hydrogenase (MvhADG) to recycle CoM, using reducing equivalents generated from the hydrogenase. However, unlike *M. stadtmanae*, the Hdr and Mvh complexes in ISO4-H5 are not predicted to be coupled to an energy-converting-hydrogenase complex [45], but rather are coupled to a F_420_-dehydrogenase Fpo-like complex to generate the membrane potential necessary for energy formation *via* ATP synthase [46, 47]. The energy converting-hydrogenase complex identified in *M. luminyensis* B10 and “*Candidatus* Methanomassiliicoccus intestinalis” Mx1-Issoire could possibly have an anaplerotic role [48]. Based on the lack of the corresponding genes, the ISO4-H5 Fpo-like complex lacks the FpoF and FpoO subunits, which in other methanogens contain the iron-sulfur centers likely responsible for interacting with coenzyme F_420_ and methanophenazine, respectively [49]_._ This is expected, as ISO4-H5 cells do not fluoresce when illuminated at 420 nm, suggesting that coenzyme F_420_ is not present in this organism. Furthermore, the genome does not contain genes for cytochrome biosynthesis, which suggests that methanophenazine is also absent. A hypothetical protein (AR505_1626) in the Fpo operon, between *fpoK* (AR505_1625) and *fpoJ* (AR505_1627) genes, is predicted to be a transmembrane protein and shares 49.5, 54.4 and 45.9 % amino acid identity to MMALV_02020 of Mx1201, TALC_00216 of BRNA1 and Mpt1_c12590 of MpT1 respectively. In addition, this gene is also located in an operon whose organization is similar to those encoding BRNA1, Mx1201, and MpT1, and is possibly a subunit of the Fpo-like complex.

ISO4-H5 is predicted to have essentially the same methane formation pathway as “*Candidatus* Methanoplasma termitum” [29] and likely pumps only one ion across the cell membrane for every two methanes formed, to generate a membrane gradient. This is in contrast *to**M. stadtmanae*, which has the same general metabolic stoichiometry but pumps two ions per methane formed [45]. Since ATP synthesis in all of these methanogens is *via* a membrane-bound ATP synthase, ISO4-H5 is predicted to have a have a much lower ATP (and growth) yield than *Methanosphaera* spp. which is consistent with the very low culture densities observed when the isolate is grown in the laboratory. However, it can be expected to have a lower threshold for hydrogen, using the same rationale proposed by Lang *et al*. (2015) for “*Candidatus* Methanoplasma termitum”. This therefore differentiates it ecologically from *Methanosphaera*, and suggests that *Methanosphaera* spp. and members of Methanomassiliiococcales, both of which occur in the rumen [24, 25], occupy different niches.

Interestingly, the cysteate synthase, cysteate aminotransferase (*serC*) and sulfopyruvate decarboxylase (*comDE*) genes required for the synthesis of CoM [50] are absent from the ISO4-H5 genome. This suggests that ISO4-H5 cannot synthesize CoM, and requires an external supply of CoM to survive within the rumen, similar to *Methanobrevibacter ruminantium* M1 [34] and MpT1 [29]. This explains the requirement for CoM supplementation in the initial enrichments of ISO4-H5 [30]. ISO4-H5 also possesses only a subset of methanogenesis marker genes: 1-8, 11, 13, 15-17 (AR505_1391, 0786, 1390, 1417, 1388, 1389, 1385, 1203, 1637, 0362, 1387, 0724, and 1386 respectively). This suggests that the remaining methanogenesis marker genes (*mmp* 9, 10, 12 and 14) are not required for the truncated methyl-reducing pathway used by ISO4-H5.

### Pyrrolysine biosynthesis

**Fig. 6:**
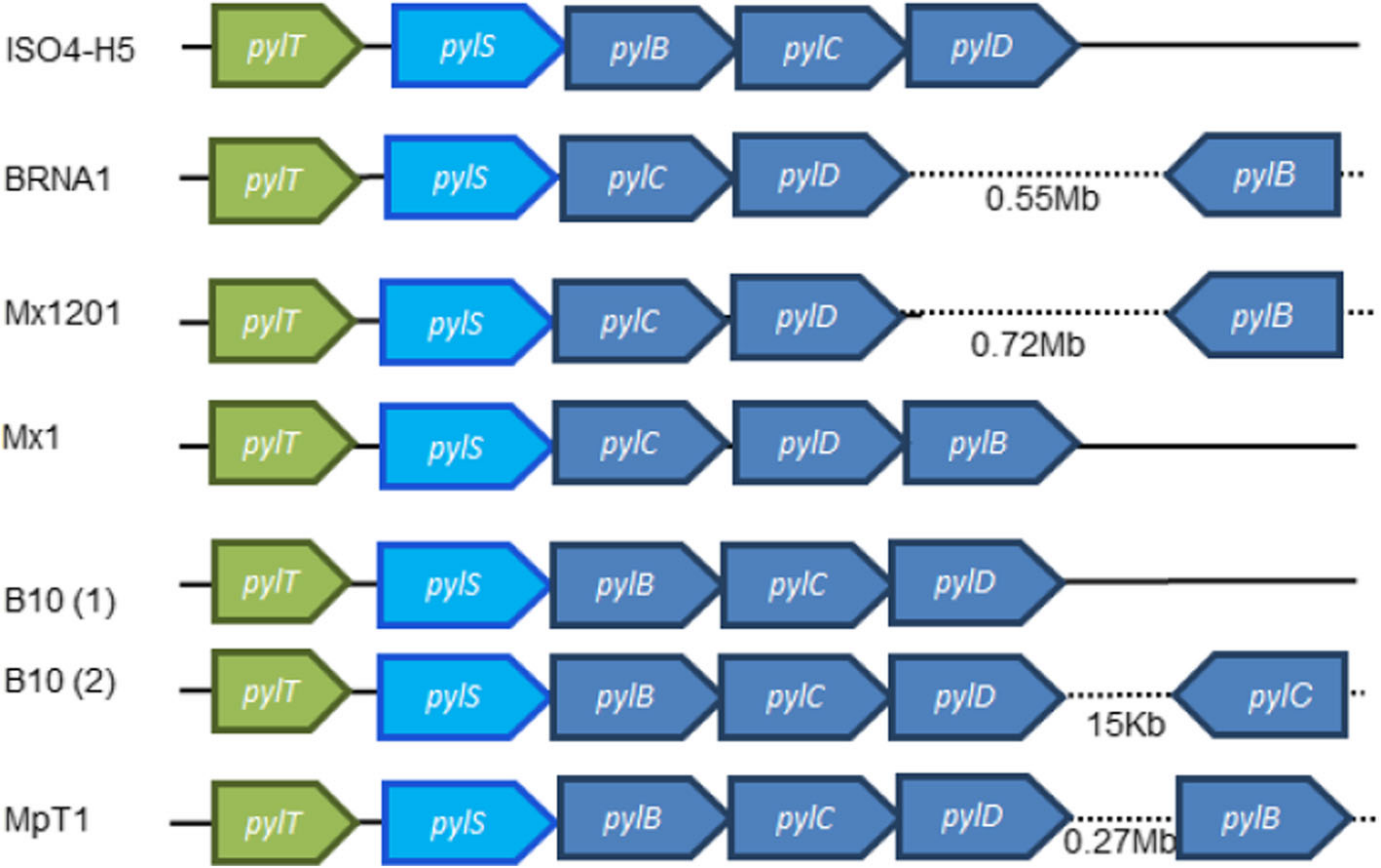
Analysis of pyrrolysine biosynthesis gene cluster in *Methanomassiliicoccales*. Gene organization of the pyrrolysine gene clusters for the six *Methanomassiliicoccales* genomes are displayed. The operon includes a pyrrolysine corresponding tRNA(CUA), *pylT*; pyrrolysine-tRNA ligase, *pylS*; methylornithine synthase, *pylB*; (2*R*,3*R*)-3-methylornithyl-*N* ^6^-lysine synthase, *pylC*; and pyrrolysine synthase, *pylD*. The strain names are given on the left-hand side of each scheme. Strain B10 has two clusters, (1) and (2), as indicated next to the strain name

ISO4-H5 possesses a complete operon predicted to encode the genes required for the biosynthesis of pyrrolysine and for aminoacylation of a transfer RNA (tRNA) to pyrrolysine (Fig. 6) [51, 52], enabling read-through of the amber stop codon, UAG. Pyrrolysine is produced from two molecules of lysine by the gene products PylBCD. Methylornithine synthase (PylB) converts L-lysine to (3*R*)-3-methyl-D-ornithine, which in turn is ligated with a second molecule of L-lysine to produce (2*R*, 3*R*)-3-methylornithyl-*N*^6^ lysine, catalysed by (2*R*,3*R*)-3-methylornithyl-*N*^6^-lysine synthase (PylC); pyrrolysine synthase (PylD) converts (2*R*,3*R*)-3-methylornithyl-*N*^6^-lysine to pyrrolysine [53]. Pyrrolysine-tRNA ligase (PylS) catalyses the aminocylation of tRNA (CUA) which itself is encoded by *pyl*T [54]. The operon organization is conserved across the *Methanomassiliicoccales* (Fig. 6), suggesting pyrrolysine use is important for members of this order. The in-frame amber codon occurs in 46 ISO4-H5 genes, including the genes encoding methylamine use; trimethylamine:corrinoid methyltransferase, *mttB* (AR505_0772); methanol corrinoid protein, *mtaC* (AR505_0952); monomethylamine methyltransferase, *mtmB* (AR505_1327, 1328); and dimethylamine:corrinoid methyltransferase, *mtbB* (AR505_1332). The amber codon is also found in the *mmp* 8 gene, a predicted nitrogenase gene (AR505_1289), an adenylate kinase gene (AR505_1784) involved in purine biosynthesis, a bifunctional phosphoglucose/phosphomannose isomerase gene (AR505_0560) involved in the last step of gluconeogenesis, two geranylgeranyl reductase genes (AR505_1433, AR505_1618) that are likely involved in cell membrane lipid biosynthesis, and the CRISPR-associated endonuclease Cas3 gene (AR505_1089) that is involved in acquired immunity against foreign DNA. Additionally, 17 genes encoding hypothetical proteins, one adhesin-like protein gene, and 10 insertion sequence elements have amber codons. Similar findings have been reported in the genomes of members of *Methanomassiliicoccales* of human origin and it has been suggested that pyrrolysine synthesis is a particular feature of this order and an important marker in the evolution of methanogenic archaea [55].

## Conclusions

ISO4-H5 has a genome size of approximately 1.9 Mb, and a genomic G + C content of 54 %, similar to the genomes of Mx1201, B10 and BRNA1. ISO4-H5 encodes the key genes and pathways required for hydrogen-dependent methylotrophic methanogenesis by reduction of methyl substrates, without the ability to oxidize methyl substrates to carbon dioxide. The wide range of methyl substrates predicted to be used by ISO4-H5 suggests it is more metabolically versatile than other methylotrophic methanogens within the rumen.

Members of *Methanomassiliicoccales* co-exist in the rumen with *Methanosphaera* spp. [24, 25, 56] and share similar substrate requirements. *Methanomassiliicoccales* are probably able to outcompete *Methanosphaera* in the rumen at low substrate concentrations, due to the lower thresholds conferred by the low ATP gain, but are probably disadvantaged when substrate concentrations are high and the low ATP yield limits their ability to proliferate. The variability of fermentation rates in the rumen associated with periods of feeding or fasting is therefore expected to give both groups of methylotrophic methanogens opportunities to grow.

ISO4-H5 appears to be reliant on the Hdr, Mvh and Fpo-like complexes for electron bifurcation, membrane potential generation and energy conservation, which is identical to what has been described in other members of *Methanomassiliicoccales*. However, ISO4-H5 is incapable of producing CoM, which suggests that ISO4-H5 has adapted to the rumen environment, where CoM produced by other methanogens would be able to supplement ISO4-H5. ISO4-H5 also lacks the genes encoding cofactor F_420_ synthesis, rendering it non-fluorescent under illumination at 420 nm. This trait has also been reported amongst other members of *Methanomassiliicoccales*, and is likely one of the key characteristics of this particular order of methanogens. However, a culture of B10 has been reported to fluoresce [57–59] and this may be consistent with B10 belonging to the deepest branching group within *Methanomassiliicoccales* [31].

The use of pyrrolysine in proteins carrying out various cellular functions suggests it is important for ISO4-H5. While pyrrolysine is important in methylamine utilisation by all members of *Methanomassiliicoccales* sequenced thus far, pyrrolysine also appears to play a role in methanol use by ISO4-H5, as the methanol:methyltransferase corrinoid protein, MtaC1, is predicted to contain a pyrrolysine in its full length protein. The use of pyrrolysine and the Fpo-like complex by ISO4-H5 adds further weight to the hypothesis that the order *Methanomassiliicoccales* is evolutionary closer to the order *Methanosarcinales*, supporting findings from a previous phylogenetic study [24]. By analyzing the genome of ISO4-H5, our knowledge of the order *Methanomassiliicoccales* has been expanded, and together with the genomes of other members of the *Methanomassiliicoccales*, will be an important resource for the development of methane abatement technologies in ruminants.

## Additional file

Additional file 1: Table S1. Associated MIGS record. (DOC 72 kb)

## Abbreviations

bp: Base pair
Cdc: Cell Division Control Protein
COG: Cluster of Orthologous Groups
CoM: Coenzyme M
CRISPR: Clustered Regularly Interspaced Short Palindromic Repeat
Fpo: F_420_ Methanophenazine Oxidoreductase
kb: Kilobase
Mb: Megabase
MCL: Maximum Composite Likelihood
Mrt: Methyl Coenzyme M Reductase II
